# Primary Epithelioid Angiosarcoma of the Uterus: A Rare Tumor with Very Aggressive Behavior

**DOI:** 10.1155/2020/5461782

**Published:** 2020-02-22

**Authors:** Nasma K. Majeed, Brian Adley, Grace Guzman, Vikas Mehta

**Affiliations:** ^1^Department of Pathology, University of Illinois at Chicago, Illinois, USA; ^2^Department of Pathology, Advocate Lutheran General Hospital, Illinois, USA

## Abstract

Angiosarcoma is a high-grade vascular tumor arising from endothelial cells of blood vessels. It represents less than 1% of the mesenchymal tumors. Uterine angiosarcoma is an extremely rare tumor with less than 25 cases reported in the literature. It usually presents in postmenopausal women as uterine mass and rarely can arise in a leiomyoma. It is included in the group of tumors of aggressive behavior and poor prognosis. Herein, we present a case of primary uterine angiosarcoma in a 56-year-old female patient with a history of fibroids presenting with pelvic pain and weight loss. Abdominal CT scan showed a large uterine mass and enlarged pelvic lymph nodes. Total abdominal hysterectomy with bilateral salpingo-oophorectomy was performed, and on histopathologic examination, it was found to be primary epithelioid angiosarcoma of the uterus. Immunohistochemical stains for CD31, keratin MAK-6, and keratin AE1/AE3 confirmed the diagnosis. In conclusion, uterine angiosarcoma should be suspected in any rapidly growing hemorrhagic uterine mass, and appropriate sampling and immunohistochemical stains should be considered.

## 1. Introduction

Angiosarcoma is an aggressive vascular tumor that mainly arises from soft tissue but can affect any part of the body. It usually affects elderly males, and the head and neck are the most common sites of diagnosis [1]. The incidence of angiosarcoma in a woman genital tract is extremely low [2]. It can involve the cervix, ovary, vagina, and rarely the uterus [3, 4]. The idea of malignant vascular tumor involving the uterus was first described by Klob of Europe in 1864 [5]. Later on in 1949, Cohen et al. used the term “hemangioendothelioma of the uterus” [6], which was later termed as epithelioid angiosarcoma.

As a term “primary” underlines, the tumor largely develops de novo with no history of radiation therapy and “epithelioid” as those tumors usually stain positive for the epithelial marker and negative for desmin and other mesenchymal markers.

## 2. Case Presentation

The patient was a 56-year-old white Caucasian female with a history of fibroids, atrial fibrillation on Coumadin, and diabetes mellitus. She presented to the hospital with two months of pelvic pain. The pain was constant and radiated into her bilateral lower back. She denied any vaginal bleeding or hematuria during this time. Menopause was at age 51. The patient was diagnosed with fibroids ten years ago and was told a year ago that the largest one was 12 cm and around the same size from her previous pelvic ultrasound, but she did not follow up regularly with the gynecologist. She also lost 42 pounds in the past year as well. No history of previous malignancy or radiotherapy was noted. On admission to the emergency department, her physical examination was unremarkable, other than abdominal tenderness. Her lab tests showed mild hypocalcemia and anemia. CT scan showed a uterine mass measuring approximately 12 × 10 × 10 cm and enlarged pelvic lymph nodes concerning for uterine cancer, and the patient was scheduled for a total abdominal hysterectomy with bilateral salpingo-oophorectomy. During the surgery, a left external pelvic lymph node was submitted for a frozen section and was reported as a malignant spindle cell tumor. The remnant of the frozen section and the tumor were all submitted for permanent sections.

## 3. Gross and Microscopic Findings

**Figure 1:**
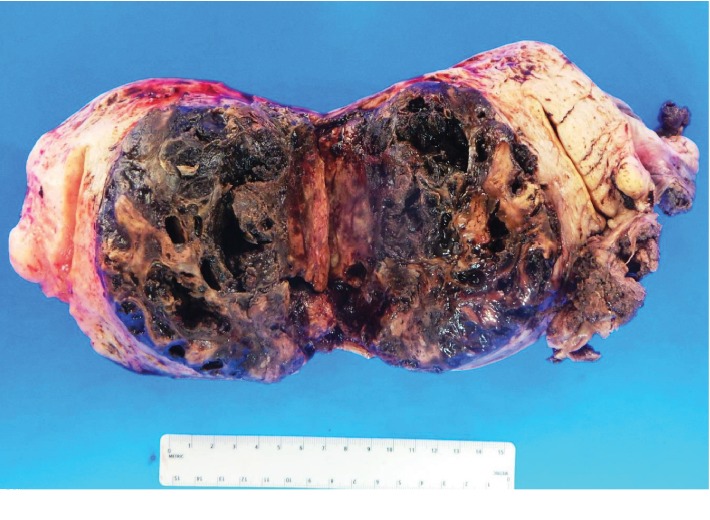
A uterus with a large tan brown mass arising from the myometrium with extensive hemorrhage and necrosis. A separate small leiomyoma identified in the uterus (arrow).

**Figure 2:**
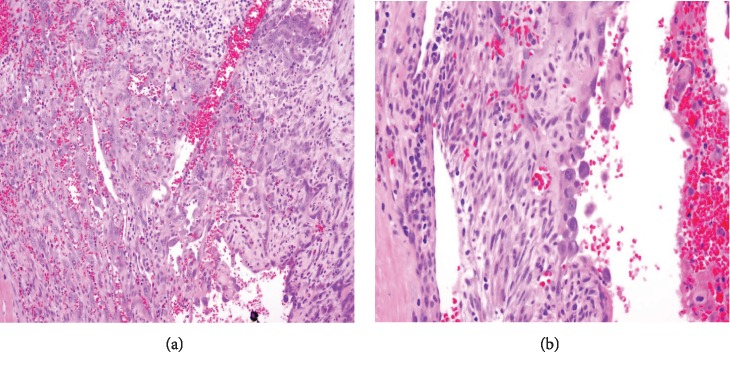
(a) A section from the uterine tumor showing vascular spaces with sheets of pleomorphic cells with abundant mitosis (hematoxylin and eosin stain, magnification x300). (b) Higher power showed malignant cells with eosinophilic cytoplasm, open chromatin, and prominent nuclei (hematoxylin and eosin stain, magnification x600).

**Figure 3:**
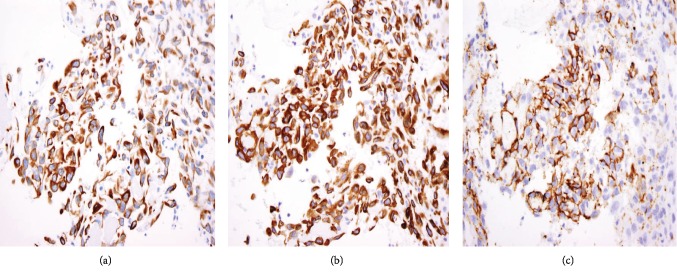
(a) AE1/AE3 stain is positive in the malignant cells (original magnification x500). (b) Keratin MAK-6 stain is positive in the malignant cells (original magnification x500). (c) CD31 stain is positive in the malignant cells (original magnification x500).

The specimen including the uterus, cervix, bilateral fallopian tubes, and ovaries weighed 1462 grams. The uterus measured 13.3 × 12.9 × 10.8 cm. Sectioning revealed a tan-red endometrium measuring 0.1 cm in thickness with two polyps measuring 1.1 and 0.9 cm in greatest dimension. The myometrium showed a protruded tan-brown mass, measuring 10.2 × 9.3 × 5.5 cm, with cystic spaces, extensive necrosis, and hemorrhage, and was seen invading deep into the myometrium. In the myometrium, there were two tan-white well-circumscribed nodules measuring 2.3 and 1.4 cm in greatest dimension. The separation between the mass and the nodules was very sharp, and there was no evidence to suggest that the mass could be arising from these nodules. Bilateral ovaries, fallopian tubes, and the cervix were unremarkable (Figure 1). Microscopic examination revealed an infiltrating tumor with extensive necrosis and hemorrhage representing more than 80% of the mass. In the nonnecrotic areas, there were irregular vascular channels lined by large pleomorphic cells with abundant eosinophilic cytoplasm, open chromatin, prominent nuclei, and mitotic figures (Figure 2). The neoplastic cells were strongly positive for CD31, keratin MAK-6, and keratin AE1/AE3 (Figure 3) while negative for desmin, smooth muscle actin, CD10, CD34, S100, MART-1, and HMB-45 by immunohistochemistry. The left external pelvic lymph node was positive for metastatic angiosarcoma involving soft tissue while the cervix, bilateral parametria, and bilateral ovaries and fallopian tubes were negative for tumor. The endometrium showed a proliferative phase with benign endometrial polyps. The two separate myometrial nodules showed benign proliferation of smooth muscle cells consistent with leiomyomata.

After the diagnosis of uterine angiosarcoma, the patient received multiple courses of chemotherapy. Two months after the surgery, the patient presented with fatigue and weakness, and her CT scan showed worsening metastatic disease with new pulmonary nodules, enlarging retroperitoneal lymph nodes and fluid in the peritoneal cavity, and the patient died three months after the initial diagnosis.

## 4. Discussion

Primary epithelioid angiosarcoma of the uterus is an extremely rare tumor. Hara et al. [7] reviewed the literature for cases of uterine angiosarcomas in 2018 and found only 24 cases including their case. Most of those patients, as in our case, were postmenopausal at the time of diagnosis.

The clinical presentation is usually uterine bleeding, anemia, and pelvic mass. Radiology is important to identify the uterine mass. However, the distinction between uterine angiosarcoma and other uterine tumor is almost impossible on imaging alone. One case report suggested that the presence of heterogeneity on T2-weighted MRI and areas of high signal intensity or what is called a cauliflower-like appearance may support the diagnosis of angiosarcoma of the uterus [8]. The distinct gross features are enlarged uterus with lobulated cross-section and extensive hemorrhage and necrosis [9]. Careful sectioning to the nonnecrotic tumor is critical to identify the sarcoma component of the tumor.

The characteristic histologic features of the tumor include a multilayer-infiltrating tumor with irregular anastomosing vascular channels and occasional tufting and papillary formation. The vascular channels are lined by large pleomorphic cells with abundant eosinophilic cytoplasm, open chromatin, prominent nuclei, and abundant mitoses along with foci of necrosis. Solid areas of pleomorphic epithelial cells or spindle cells can be identified as well [10]. Immunohistochemical stains are very important to confirm the diagnosis of uterine angiosarcoma especially for the high-grade tumor. The malignant cells are usually positive for CD31, factor VIII, ERG, and cytokeratin and have a variable expression for CD34 [11]. CD31 is the most specific for this tumor. In our case, the tumor cells exhibit positivity to CD31, keratin MAK-6, and AE1/AE3 consistent with primary epithelioid angiosarcoma.

The pathogenesis of uterine angiosarcoma is not very clear. Although majority of the cases of primary uterine angiosarcoma arise de novo, seven cases were found to be arising from leiomyomata [7, 12]. The mechanical pressure from the leiomyoma can cause abnormal vascular malformation and subsequent malignant transformation that may play a role in the pathogenesis of these tumors [13]. In our case, the patient had a long history of fibroids, and her last ultrasound showed a huge leiomyoma measuring more than 10 cm; however, most of the tumor tissue was necrotic with vascular channels and pleomorphic cells in between with no evidence of benign smooth muscle tumor. Moreover, the two separate leiomyomata that we identified were small and far away from the main tumor. We think that the primary uterine angiosarcoma in our case is probably de novo that had been mistaken as a fibroid in the previous ultrasound; however, the possibility of angiosarcoma completely replacing the leiomyoma over time cannot be excluded.

Some authors suggested that molecular analysis can be a useful diagnostic tool in challenging cases. Suzuki et al. [14] identified breakages at three loci: YWHAE (17p13), FAM22A (10q23), and FAM22B (10q22), in the case of uterine angiosarcoma. The molecular-genetic alterations, in this case, appear similar to those of endometrial stromal sarcoma suggesting that those genes may play a role in the development of the uterine angiosarcoma. It may also suggest that high-grade endometrial stromal sarcoma with those mutations may show angiosarcomatous transformation. However, due to the rarity of this tumor, there is not enough data to support this hypothesis. Other genetic mutations like MYC, CIC, FLT4, KDR, and PTPRB mutations have been described recently in cases with angiosarcoma. However, there is no enough evidence that there is a specific molecular profile to differentiate between primary epithelioid angiosarcoma of the uterus and other types of angiosarcoma [15].

The management usually includes total abdominal hysterectomy with bilateral salpingo-oophorectomy followed by neoadjuvant therapy [16, 17]. The most important prognostic factors are the size of the tumor and presence of an extrapelvic tumor at the time of the diagnosis [18]. Unfortunately, uterine angiosarcoma is a highly aggressive tumor, and the lymph node and distant organ metastases are very common, and more than half of the patients that have been reported died in less than one year after the diagnosis [19, 20].

In summary, we report a rare case of primary uterine angiosarcoma in a 56-year-old female. Although this tumor is very rare, it should always be considered in any patient with necrotic and hemorrhagic uterine tumor, and careful examination is necessary to identify any sarcomatoid component as these tumors usually have a very aggressive behavior and are associated with poor prognosis.
